# Aligning SDG 13 with South Africa’s development agenda: Adaptation policies and institutional frameworks

**DOI:** 10.4102/jamba.v14i1.1155

**Published:** 2022-03-18

**Authors:** Dumisani E. Mthembu, Godwell Nhamo

**Affiliations:** 1 Department of Environmental Sciences, University of South Africa, Johannesburg, South Africa; 2 Institute for Corporate Citizenship, University of South Africa, Pretoria, South Africa

**Keywords:** SDGs, adaptation, resilience, stakeholders, alignment, climate change

## Abstract

The alignment of the Sustainable Development Goals (SDGs) with national development agendas has gained traction since the ratification of the 2030 Agenda for Sustainable Development in September 2015. This article investigates how South Africa has aligned the climate action SDG (SDG 13) with its national development agenda, with an emphasis on adaptation policies and institutional framework. This comes against a background where the country has been accused of bias towards mitigation policies that were trigged by the Long-Term Mitigation Scenarios in 2007, which could have quickened mitigation responses to SDG 13. The data were generated through the use of three key methods, namely key informant interviews (*n* = 21), an online survey uploaded on an online platform called QuestionPro and a realised sample of 103 completed surveys. Furthermore, relevant policy documents were analysed from a critical discourse perspective. It emerged that South Africa has policies and strategies in place to respond to climate change adaptation within the context of SDG 13. However, while policies are in place, they have not translated to real change on the ground and therefore have not enabled the country to have adequate climate change resilience. The policies have not been translated into concrete actions; there are knowledge gaps in adaptation, poor leadership and lack of clear vision for adaptation and poor coordination. Institutions are scattered, with uneven capacity across sectors and different spheres of government; and weakest at the local government level. It also emerged that mitigation was prioritised for a while over adaptation, with a lack of funding and general awareness. The study recommends that adaptation measures should not be undertaken in isolation, instead, it should be addressed within the context of other programmes such as disaster risk management and sustainable development.

## Introduction

According to Arndt et al. (2012), climate change presents a highly complex challenge for developing countries. There is a growing acknowledgement of the effects of climate change across various sectors of life and the economy. This is because climate change has brought an additional layer of burden to the already economically, socially, environmentally and politically challenged economies. The climate action Sustainable Development Goal (SDG 13) of the 2030 Agenda for Sustainable Development is explicit concerning the need to ‘strengthen resilience and adaptive capacity to climate-related hazards and natural disasters in all countries’ (United Nations 2015:23). Even though climate change is unprecedented, global climate policy has not been without problems. There has been an overemphasis on climate mitigation at the expense of climate adaptation. As a result, critical issues relating to climate resilience that fall within the adaptation realm and with severe impacts on economic sectors such as agriculture, health and water appear to have remained peripheral in global discourses.

Whilecontention by Otto et al. (2015), on the slow progress on mitigation is observed, there is overwhelming evidence that supports the view that a global drive on climate change initially was a mitigation agenda. Saab (2016) was of the view that the bias in favour of mitigation can be traced from the earlier reports of the Intergovernmental Panel on Climate Change (IPCC), the texts of the United Nations Convention on Climate Change (UNFCCC) and Kyoto Protocol. The author indicated that the objectives of the UNFCCC and the Kyoto Protocol prove this point. The author further argued that the few general references to adaptation in these instruments do not appear to go far enough because they are vague, broad, open-ended and lack specificity. However, the recent developments are encouraging. Africa’s Agenda 2063 has committed to address climate change by prioritising adaptation in all its actions while participating in global efforts for climate change mitigation that enhance and strengthen the policy direction for sustainable development on the continent (African Union Commission 2015). If adaptation is prioritised, it will make sure that adaptation measures do not become peripheral and it is nice to have intervention in the continent.

Given the foregoing discussion, this article is dedicated to investigating how South Africa has aligned the climate SDG with its national development agenda following the proclamation of the 2030 Agenda for sustainable Development in 2015 (United Nations 2015). The focus is on climate adaptation and resilience and related institutions. To this end, the research question is spelt out: Which policies and institutions are dealing with climate change adaptation capacity and resilience building, and what are the provisions of such policies in South Africa? Similarly, an objective is crafted to determine policy coverage and institutional spread regarding the addressing of climate change adaptation capacity and resilience building in South Africa while endeavouring at the same time to contribute to knowledge. For instance, Creswell (2014) regarded the need to improve policy, decision-making and practice as critical considerations to contributing to knowledge. In this context, given the focus of this article that examines the policy coverage and institutional spread relating to climate change adaptation in South Africa, the call by Creswell (2014) remained relevant. In this regard, the authors are of the view that while a lot is written about climate change policy in general in South Africa, the extent to which this has been analysed from an implementation and institutional perspective remains limited. Therefore, this article seeks to contribute to knowledge specifically in this area in order to improve policy, practice and even decision-making.

The article begins by discussing the politics that has engulfed climate change adaptation in general both globally and domestically. This is followed by a reflection on some of the factors that hamper adaptation efforts. The article then moves to research methodology, highlighting the research findings and concluding with policy recommendations particularly for the government.

## Literature review

### The politics of climate change adaptation

The underlying assumption of this article is that the politics of climate change adaptation particularly at a global level has not helped to advance the adaptation agenda at national level. This dynamic, coupled with barriers to adaptation has hindered the adaptation efforts nationally, including in South Africa. Masters (2011), as well as Simonet and Fatoric (2016), had criticised the framing of the concept of adaptation in international climate change discourse. Adaptation is portrayed as a local issue that does not require global policy discourse. Even though Masters (2011), acknowledged that the contestations around adaptation have heightened tensions in international negotiations, it is disappointing that Africa’s approach to adaptation has been largely perceived to be reactive. This could be an indication that adaptation has not been a mainstream issue in climate change response. Given the foregone, and the changing policy terrain in South Africa, this work will get into an in-depth analysis of South African policy as it relates to the climate change adaptation agenda.

Laves et al. (2014) recalled that during the early 1990s, adaptation measures were often viewed with scepticism as an indication that mitigation policies could not work. For instance, Al Gore associated adaptation with laziness that side-tracked efforts and resources away from the core issue of mitigating impacts (Laves et al. 2014). As observed by the authors, it is indeed heartening that adaptation has been accepted as a valid and critical intervention for addressing the unavoidable impacts of climate change. Recently, there has been a growing body of knowledge focusing on auditing how African countries have been reporting on adaptation in their National Communications with articles on livestock and fisheries (Muchuru & Nhamo 2017).

Be that as it may, this cannot take away the fact that there was a missed opportunity to adapt to climate change. Similarly, given the prioritisation of mitigation measures globally, it can be concluded that most countries at the national level may have followed this trend where adaptation was not given the urgency it deserves because national policies are by far influenced by trends in policies at a global level. With the growing consensus about the need to adapt, recent discussions are paying more attention on how to operationalise adaptation in policy practice (Biesbroek et al. 2011).

Linked to the growing consensus about the need to adapt, Simonet and Fatoric (2016) posited that since 2001, adaptation was given prominence in IPCC assessment reports, signalling that the urgency to focus on adaptation strategies was no longer inevitable. The fourth assessment report (AR4) released in 2007 confirmed that adaptation remained the only available and appropriate response to impacts that were already being felt and those that would arise in future (IPCC 2007).

Masters (2011) highlighted the urgency of adaptation in Africa within the context of sustainable development. The author warned that the failure to address adaptation could threaten to reverse the gains of the Millennium Development Goals (MDGs). The author further argued that an understanding of adaptation needs to go beyond environmentalism and highlight true socio-economic and developmental impact of climate change on issues such as food, health, water, poverty and migration. Given the sensitivity of agriculture, Mccarl, Thayer and Jones (2016), posited that this in turn will influence agricultural productivity and water needs. As such, the authors recommended that more efforts may be needed in adaptation in the foreseeable future given the pace at which the climate is projected to change.

### Factors perceived to be hindering adaptation measures

While increasing voices for more adaptation measures are warranted, such measures may be hindered by the existence of the plethora of barriers to adaptation. Although there are challenges with adaptation in general, Simonet and Fatoric (2016) acknowledged that the literature on adaptation to climate change is emerging as a distinct field of science even though there is still a challenge of vagueness and unclear definition of adaptation and its various interpretations. This could hinder implementation. Furthermore, there is still a research gap regarding the availability of relevant knowledge to sufficiently inform climate change adaptation policymakers (Laves et al. 2014). This is attributed to the paucity of research reporting on the effectiveness of implemented adaptation strategies and the fragmented nature of the adaptation research community and the disparity between disciplinary perspectives.

Evidence suggests that barriers tend to differ from project to project and from area to area. Biesbroek et al. (2011) maintained that because barriers are highly context-specific, it is challenging to compare and difficult to use for a more generalised understanding (Biesbroek et al. 2011). For instance, experience from Netherlands, which is considered to be among the frontrunners in climate change research and policy, provides a comprehensive and instructive perspective on barriers to adaptation. Lessons from the Netherlands highlighted the following barriers: (1) uncertainty, (2) the high cost of adaptation measures, (3) fragmentation, (4) unavailability of data, (5) lack of national focus to climate change, (6) pre-existing cultural beliefs and (7) inadequate appreciation of the possible effects of climate change.

There are also numerous underlying causes of these barriers. Barriers caused by conflicting timescales were found to be the most important group of barriers. This is critical because climate change is one issue that has to compete with other critical issues for an already limited amount of political attention; issues that are more pressing in nature, whose impacts are more immediately felt or have more visible short-term results than adaptation effects, which are long-term (Biesbroek et al. 2011). As a result, the conflicting timescales makes it more challenging to integrate adaptation in new and existing policies and practices.

The availability of resources is an important factor in adapting to climate change, inadequate resources or the inaccessibility thereof can admittedly be a major barrier to climate change adaptation. Saab (2016) had decried the fact that private sector involvement is not properly defined; at a time when it is felt adaptation efforts should not be an exclusive responsibility of government. One, however, should be cautious when dealing with the issue of resource availability as it is not a silver bullet, as it may seem. Generating adaptive capacity may be one thing but mobilising already existing adaptive capacity and turning it into effective adaptive machinery may be a different challenge altogether.

Another group of barriers worth scrutinising is social barriers. Social barriers tend to be widespread and difficult to manage. Paradoxically, they are integral in determining the level of success of adaptation efforts. For instance, in Australia, the community rejected the proposal to drink recycled water, which highlighted the significance for policymakers to gain the community confidence before applying adaptation measures (Laves et al. 2014). It is for this reason that an emerging priority area of research in this field is communication to deal with perceptions, attitudes, ethical beliefs, norms, emotions and trust needed (Laves et al. 2014). This will increase public consciousness and awareness and the role of communities in adaptation.

Oberlack (2017) highlighted the role of institutions in providing an enabling environment for adaptation. Institutional crowdedness and institutional voids present a different set of challenges. For instance, lack of enabling institutions and policies, guidelines and legislation can make communication and facilitation among stakeholders more challenging, particularly where there are no common guidelines, principles, values or standards about adaptation (Oberlack 2017).

By contrast, institutional crowdedness manifests itself through the existence of a plethora of institutions, which influence the decision-making process on climate change adaptation. This may result in unnecessary competition among institutions, cause confusion about roles and responsibilities and lead to different approaches and perceptions about the problem (Oberlack 2017). Clearly, such a situation would be untenable and it could even cause confusion on how the problem should be solved. As this research was unfolding, critical attention was made to identify some of the challenges highlighted herein for South Africa.

Laves et al. (2014), observed that many climate change adaptation efforts are being integrated through a policy that may not be recognised as such by adaptation experts. Consequently, its benefits are realised unintentionally as by-products of non-climate change issues such as disaster risk management, sustainable development, cost savings and efficiency measures (Laves et al. 2014). Arguably, this diminishes the role of adaptation in policy and broader political discourse. There is also a problem of maladaptation.

According to Mccarl et al. (2016), maladaptation occurs when adaptation actions of one party have unintended consequences on other parties or areas. Lack of proven adaptation experience increases the potential for unforeseen and undesirable outcomes (Laves et al. 2014). Given all these challenges, one can sympathise with Halvorssen (2008) who maintained that climate change effects demand a strong action and international cooperation comparable to the Marshall Plan that was put in place after the Second World War. Such strong action must be supported by multidisciplinary tools and instruments to ensure an effective and enhanced response.

## Materials and methods

The study followed the evaluation research principles, which are usually used in big organisations, such as the government, often with a purpose to assess the effectiveness of a new programme or policy so that the necessary improvements can be made. Accordingly, Creswell (2014) highlighted that evaluation research is performed primarily to develop a thorough analysis of a particular case, in this instance, the extent to which South Africa has aligned adaptation measures and related institutional mechanisms to its development agenda. It has been suggested, at least by Creswell (2014) as well as Leedy and Ormond (2015) that the best way to achieve this is through the use of mixed-method approach (MMR) which combines the use of various research methods. Hence, it is also called method triangulation.

Consistent with the proposition by Creswell (2014) to tease out the research question and its objective stipulated in the introduction, several methods were utilised in generating data. These methods included an online survey, key informant interviews, critical discourse and document analysis. Respondents were asked a series of questions relating to the adequacy of climate change policies as called for by Leedy and Ormrod. In this regard, the respondents were asked among others (1) if South Africa has policies in place designed to respond to climate change adaptation, and if those policies adequately address climate adaptation challenges faced by it, (2) to highlight the available institutional capacity that is in place to implement the different climate change adaptation sectors, (3) if the sectors that must be prioritised, (4) to rate the importance of adaptation in South Africa, including on whether or not there is adequate emphasis on adaptation and the success of those adaptation measures (5) to identify the vulnerable sectors that require urgent attention, (6) if coordination of various institutions involved in adaptation measures are effective, (7) to highlight the main adaptation barriers facing South Africa and (8) an overall assessment if climate change adaptation is neglected or not.

Twenty-one key interviews were held based on a pre-developed questionnaire. The interviews helped the researcher to obtain first-hand information concerning the research objectives of the study. Among some of the key informants were government officials involved in drawing up national policies related to climate change (adaptation) and sustainable development, as well as global negotiations. The interviews have the advantage of assisting and enabling the researcher to connect with respondents and therefore gain their cooperation. Even though they are time-consuming and expensive, interviews tend to yield the highest response rates (Leedy & Ormrod 2015).

According to Leedy and Ormrod (2015), there are three widely used techniques that can facilitate the quantification of complex phenomena. These are rating scales, checklists and rubrics. The rating scale techniques were built in the online survey mailed to approximately 700 potential respondents with a realised sample of 103. For example, the Likert technique is relevant for studies that may be perceived by some as controversial by asking questions that are not normally asked. According to Kandasamy et al. (2019), Likert scale is the most commonly used instrument to gather data from people in a survey based on the level of agreement or disagreement with a particular proposition presented to the respondents. This study raised issues that had to be opinion rated and that might be perceived to be critical of government policies. Accordingly, the respondents were asked to give perceptions regarding the extent to which they agreed or disagreed with a particular statement. To answer the question, the respondents were required to choose from two opposing extremes of strongly agree and strongly disagree and other common and acceptable Likert scale responses.

Furthermore, to probe underlying meaning, semantic differential and categorical response techniques were employed. The semantic differential technique is useful to measure indirectly how the respondents feel about a particular issue and to compare responses among respondents. The semantic technique allows respondents to express feelings by ratings with respect to opposing concepts. The categorical responses technique was used for questions deemed to be of sensitive nature. Some of the questions were designed to obtain information indirectly using this technique. This technique allows the respondents to place themselves in categories rather than to give exact answers. The key to the effective application of this technique is to ensure that all possible options are covered and guarded against overlaps between categories.

The selection of respondents was undertaken simultaneously for both the online survey and the interviews before data collection commenced. Furthermore, all respondents who participated in the online survey and interviews were asked to respond to the same broad set of pre-determined questions as discussed earlier. As policy on climate change is the responsibility of national government departments, the majority of respondents, particularly key informants were purposefully selected from these departments. These included among others, the Department of Environmental Affairs, Department of Energy, Department of Planning, Monitoring and Evaluation, Department of Science and Technology, Department of Agriculture, Forestry and Fisheries and the Department of International Relations and Cooperation. Respondents (middle to senior management) were purposefully selected from mainly the environmental and economic clusters of government departments. Furthermore, climate change adaptation policy and related documents were also gathered. This permitted both the document and critical discourse analysis as a method (Vogt, Gardener & Haeffele 2012).

In addition to focusing on national government departments, the online survey technique was emailed to state-owned entities (SOEs) that perform government mandate related to climate change and sustainable development, research organisations and non-governmental organisations. The SOEs included Electricity Supply Commission (ESKOM), South African National Biodiversity Institute (SANBI), Council for Scientific and Industrial Research (CSIR), South African Weather Service (SAWS), South African Environmental Observation Network (SAEON), Applied Centre for Climate and Earth Systems Science (ACCESS), South African Energy Development Institute (SANEDI), National Cleaner Production Centre (NCPC), Statistics South Africa (StatsSA), National Disaster Management Centre (NDMC), stakeholders in the renewable energy industry, academia among others.

As indicated earlier, 103 respondents finished the online survey, which had been loaded onto the QuestionPro platform. Data collection through the online platform commenced from December 2018 to August 2019, while the interviews were held between February and September 2019. The 21 key informants who took part in the interview were coded from 1 to 21 to ensure anonymity and confidentiality.

Pertaining to ethics, the study ensured that respondents were provided with adequate information about the study, including the risks so that they could make an informed decision of whether or not they wished to participate in the study. Furthermore, the researchers ensured that the integrity and quality of the research are not compromised. Hence, the need to ensure the well-being of the participants in the study. One way of achieving this was to guarantee the privacy of participants in the study by protecting their identity and ensuring that the information obtained always remains secret and is stored securely at all times. Prior to participating in the study, the respondents were provided with the participant information sheet that was approved by the General Research Ethics Review Committee of the College of Agriculture and Environmental Sciences (University of South Africa).

This provided them with all the necessary information about the study, why they had been selected to participate in the study and what was expected of them. It further informed them of their rights, how the data would be handled and used, assured them that their privacy and anonymity would be protected and that they would be required to consent in writing to participate in the study. Encryption of specific details was employed where it was relevant to protect the identities of respondents so that colleagues from the same organisation could not identify the participants from information about the study. As such, participants were asked to indicate which information should be kept secret. At an institutional level, permission was sought from organisations that may be affected by the study, particularly the Department of Science and Technology, the Department of Environmental Affairs and the Department of Energy. All data collected were used for the sole purpose of achieving the research objectives.

Of the respondents who took part in the online survey, 51% were male while 46% were female. With regard to the racial composition of the sample, 56% were black, 29% white and 14% fell in other racial groups. A total of 95% of respondents were employed, the majority of whom (31%) held middle management positions, 26% in senior management and 11% in executive management. Similarly, 90% of the surveyed respondents possessed honours degree and above, while 10% had diploma and undergraduate qualifications.

Accordingly, 45% of respondents were very familiar with climate change management policies in South Africa, while 46% were moderately familiar and only 9% indicated that they were not familiar. With regard to sustainable development, overall, 95% of respondents were very familiar with sustainable development strategies in South Africa. Only 5% indicated that they were not familiar with sustainable development.

## Presentation and discussion of findings

### Policies and strategies designed to respond to climate change adaptation

Boyd et al. (2009) explained that developing countries are particularly most vulnerable to climate risks. South Africa as a developing country in Africa is no exception to this phenomenon. When asked if South Africa has policies in place to deal with climate change adaptation, there was a high degree of agreement among respondents that South Africa has policies and strategies in place designed to respond to climate change adaptation. A total of 78% of respondents surveyed agreed that South Africa has developed good policies for climate change adaptation.

**FIGURE 1:**
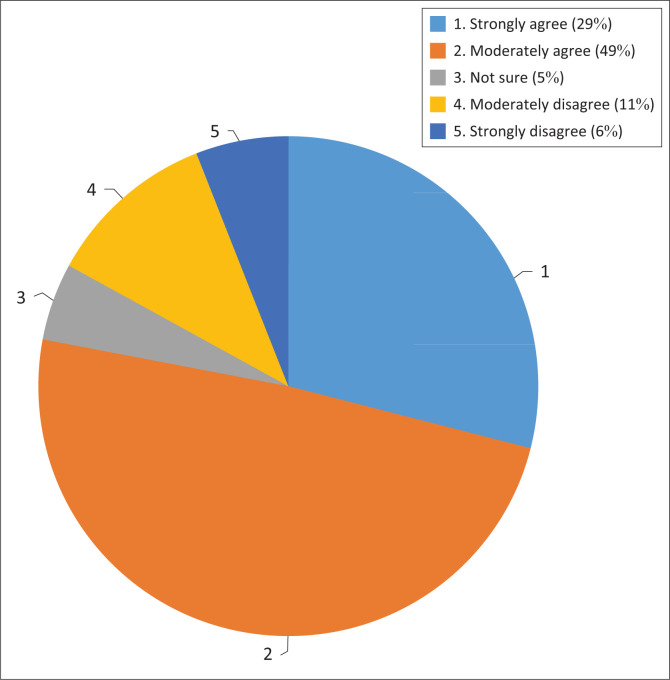
Availability of policies to respond to climate change adaptation.

Given that climate change adaptation is largely seen as a local issue globally, it can be inferred that developing countries are able to exercise much more direct policy control with respect to adaptation than with respect to mitigation. In addition to the policies and strategies that were identified, the *Draft National Adaptation Strategy* that was published on 06 May 2019 for public comment was cited as the latest addition to a suite of strategies to deal with adaptation that has been developed. The *Draft National Adaptation Strategy* has since been approved for implementation by Cabinet in August 2020. This Strategy is also intended to be the National Adaptation Plan under the Paris Agreement. Figure 1 demonstrates graphically how the respondents reacted to this question.

The respondents were asked if they thought the policies adequately address climate change adaptation challenges faced by the country. What was notable was that even though the respondents felt that the policies are in place, 39% of the respondents were of the view that such policies do not appear to have influenced climate change adaptation challenges that are faced by South Africa. Among other reasons, they perceived climate change as a moving target that requires strong implementation.

On the contrary, the respondents repeatedly asserted that the policies have not been translated into concrete actions. Failure to translate the policies into concrete action has seemingly resulted in the laxity of implementation in general which the respondents felt was necessary. It is also for this reason that the policies were found to be superficial and only good on paper with limited impact on the ground (Respondent 13).

**FIGURE 2:**
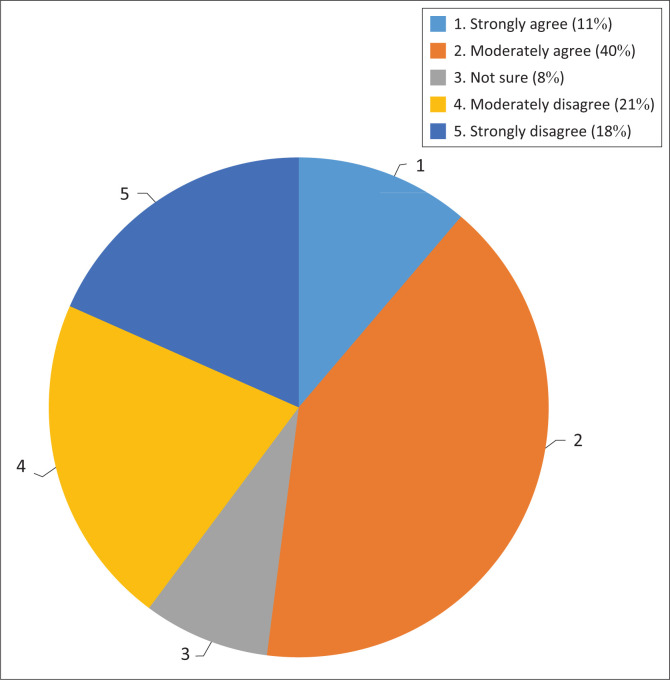
Policies adequately address climate change adaptation challenges.

Fischer-Smith (2018) observed that in Ukraine, mechanisms were put in place on paper but were not given sufficient resources to accomplish their goals. The respondents also highlighted that the scale of adaptation is enormous, ironically, there seems to be little appreciation of the scale and enormity of the challenges that South Africa faces regarding adaptation (Respondent 1). Figure 2 shows how the respondents expressed their views on whether or not the policies adequately address climate change adaptation challenges.

Another perspective that emerged from Respondent 19 that is linked to poor implementation relates to what was termed as ‘gaping-holes in knowledge base’. This did not necessarily mean new knowledge but rather critical knowledge that would complement existing knowledge, for example, on predictions. This came as a result of a recognition that South Africa has the ability to make good projections. However, it was contended that very little if any appears to have come out in terms of the second-tier applications where such projections are applied to a specific sector domain such as water, agriculture and others.

It was suggested that such analysis of projections to those domain levels would provide South Africa with empirical evidence on how the respective sector domains would be affected in the short-, medium- and long-term. This would also assist the country in determining exactly what it needs to be adapting to. Laves et al. (2014) appears to share this sentiment. According to the authors, despite the growth in adaptation research, there is still a knowledge gap in understanding the information required and used by policymakers when making decisions.

Furthermore, Laves et al. (2014) contended that even though the first and second generation of adaptation research have provided significant insights into adaptation processes, however, they have proven to be unsuitable to help decision makers to select types of adaptations that might best suit particular circumstances. Hence, the authors have advocated for a third-generation research than mainstream theory, policy and practice. As a result of the knowledge gap, as illustrated in the literature review, it was suggested that South Africa has been found wanting on many fronts. Respondent 19 decried the lack of a clear sense of where the country wants to go when it comes to adaptation. It was argued that this has resulted in a weak and ill-informed vision that does not correlate to the development objectives of the government. Fischer-Smith (2018) highlighted the impact of leadership on policy processes as a critical element for successful implementation.

The respondents indicated that the lack of vision also manifests itself in government departments that are important for adaptation that do not appear to be fully engaged with adaptation issues and challenges (Respondent 15). Hence, 54% of the respondents felt that while policies are in place, they have not translated to real change on the ground and therefore have not enabled South Africa to have adequate climate change resilience. This was raised partly because Respondent 6 felt that South Africa still experiences incidents that have happened in the past and with similar consequences as if there are no adaptation measures in place. Similarly, it was felt that past experiences do not seem to have influenced how things are carried out when it comes to adaptation responses. Having said that, the respondents felt that some sectors should be given more attention when it comes to adaptation interventions (Table 1).

**TABLE 1:** Sectors that must be prioritised for adaptation.

Sectors that must be prioritised to meet the desired policy objectives for adaptation		
Water and catchment management services	Biodiversity	Ecological infrastructure and services
Ecosystem management services	Agriculture	Early warning systems
Human safety	Transport sector and/or infrastructure	Fisheries
Methodologies for estimating the adaptation costs	Institutional capacity to deliver on actions	Processes that define adaptation objectives

One of the areas raised here that need urgent attention relates to agriculture. According to Arndt et al. (2012), when considering the implications of climate change, a natural place to start is the agricultural sector. The authors contend that this is true especially where (1) agriculture remains a critical contributor to the economy, (2) agriculture is a key driver for employment, (3) and food represents a high share of household consumption. Similarly, they concur with previous studies that suggests that climate change can have major implications for long-term infrastructure planning, which may result in the escalation of maintenance and construction costs. Such escalation of costs could in turn hinder the development of key economic infrastructure (Arndt et al. 2012).

Although these sectors came up, Respondent 19 was of a view that South Africa needs to first and foremost define its economic and development interest. It must also understand how it is impacted by climate change and how those economic interests affect the socio-economic priorities. Based on that, it would then be easy to define what needs to be prioritised and funded. While it was felt that funding could be an issue, it was suggested that it could easily be sourced provided a compelling case is advanced for resources to be made available.

### Institutional capacity to implement climate change adaptation across sectors

The challenges discussed earlier about the laxity of implementation may be attributed to the issue of capacity to implement policies and programmes. This is a serious issue that cannot be ignored to effectively implement policies and strategies. Fischer-Smith (2018), suggested that the health of a delivery institution is closely linked to the efficiency and effectiveness of implementation. Furthermore, the author cautions that even well-designed policies can only do so much if institutional conditions are not conducive. Hence, the author argues that policies are less durable than institutions.

**FIGURE 3:**
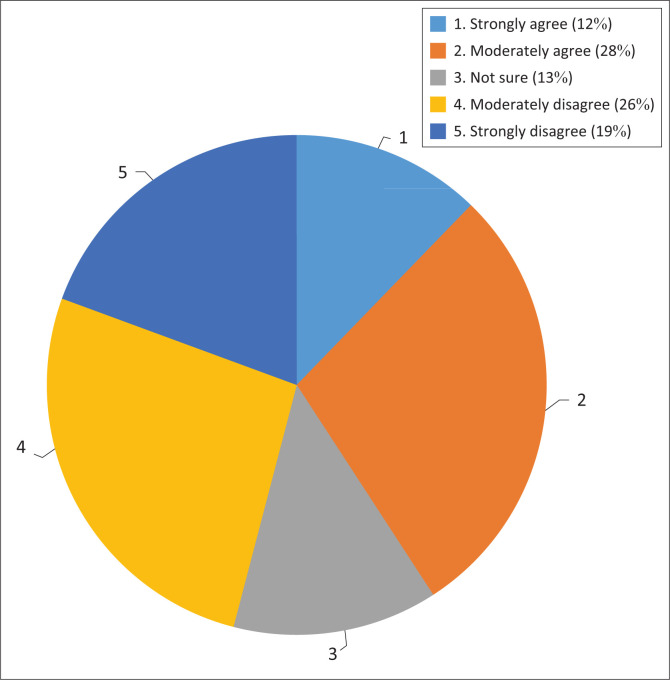
Availability of institutional capacity to implement adaptation measures.

Put differently, it does not help to have good policies when there are no institutional mechanisms or weak institutional capacity to derive value from those policies. When asked if they thought that South Africa has institutional capacity to implement the different climate change adaptation sectors identified in the policy there was a mixed reaction to that question as shown in Figure 3. A total of 46% of the respondents surveyed felt that institutional capacity to implement policies was lacking while 40% felt that South Africa has requisite institutional capacity needed to implement its climate change policies.

A total of 46% respondents who disagreed felt that despite the best intentions and good-will that South Africa has shown; the reality is that South Africa does not appear to have built adequate institutional capacity. They argued that even where capacity exists, it appears to be scattered and uneven across sectors and different spheres of government. This is something that Marquardt (2017) cautions against because of the recognition that issues such as regulatory competencies, financial capacities and even inter-jurisdictional conflicts may shape environmental governance.

Worryingly, there was a concern that there appears to be a complete lack of capacity at the municipal government level. The lack of capacity was attributed in part to adaptation being a ‘poor cousin and a late comer’ compared with mitigation (Respondent 8). Faling, Tempelhoff and Niekerk (2012) wrote extensively about the range of capacity constraints that are faced by local government, including lack of understanding by officials of climate change science and its implications at the local level. While capacity and resources may be a hindrance in fostering policy implementation, Marquardt (2017) argued that actors must be able to utilise the resources at their disposal including financial and knowledge resources. Among the institutions that were cited include the department responsible for environmental affairs, sector departments that are required to have sector adaptation plans, SANBI, CSIR, SAWS, Agricultural Research Council (ARC) and Water Research Commission (WRC).

It was recommended that as South Africa strengthens its institutional capacity, it would also have to define the roles of different players. Fischer-Smith (2018) cited among others the unclear chain of command and multiple actors as possible barrier points to effective implementation. This somewhat confirms what was highlighted in the literature review that institutional crowdedness and institutional voids may present challenges of their own; hence the imperative to ensure that they are well thought through, managed and coordinated. The respondents also cautioned that while the department responsible for environmental affairs is critical to provide leadership, implementation should not be left to it alone as this would not be effective. The respondents emphasised that other sectoral departments should play their rightful role at a sector level to monitor and enforce implementation.

Clearly, implementation cannot happen in a vacuum unless there is effective coordination. When asked about the effectiveness of the coordination of various institutions that are involved in climate change adaptation, the respondents viewed the institutional coordination as being largely ineffective and uneven across sectors and spheres of government. Exceptional pockets of excellence at national level, KwaZulu-Natal and the Western Cape provinces were highlighted (Respondent 6). According to Galvani (2018), uneven implementation can be attributed to varying socio-economic characteristics, which often result in a disparity in implementation performance.

Among the coordination mechanisms that were cited included the MINMEC^1^ and the MINTEC^2^ that are supported by various technical working groups and committees, government cluster coordination, Intergovernmental Committee on Climate Change (IGCCC) and National Climate Change Committee (NCCC) at national level. Respondent 7 indicated that in provinces, they now have provincial climate change forums including in some local municipalities. Outside government, civil society and non-governmental organisations are also doing a lot of work, which calls for collaboration with government and within the government to break the silos.

The respondents decried the inherent disjuncture and lack of coherence at what happens at the different spheres of government. Hence, each sphere of government appears to be doing its own thing with limited communication and collaboration with other spheres sectors. Marquardt (2017) highlighted coordination across jurisdictional levels as a crucial necessity. While the respondents attributed poor coordination to lack of leadership and effective coordination; Kennedy and Chen (2018), attributed that to the tension that emanates from the administrative tug of war between government goals and local government.

With this in mind, Respondent 19 argued that what seems to be the real problem is not necessarily lack of ability to convene different sector players, but rather the thought leadership that is lacking to ensure that these structures can be used much more effectively. Therefore, it was insinuated that ineffective coordination is not because of the lack of structures but rather it is because of the lack of knowledge and thought leadership to achieve it, hence coordination largely remains reactive and not proactive

### Key barriers to adaptation

**FIGURE 4:**
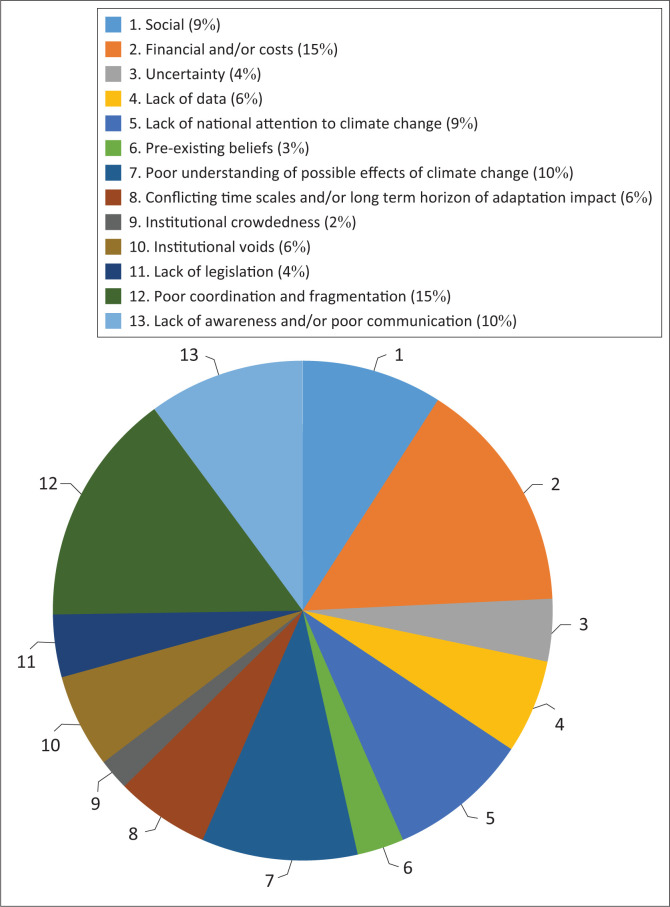
Barriers that present most challenges to adaptation.

The respondents were asked to identify the five most important barriers they thought presented the maximum challenge for South Africa in responding to climate change adaptation. Figure 4 depicts how the respondents reacted to the question. It shows that financial and/or costs, lack of awareness and/or poor communication, poor understanding of possible effects of climate change, lack of national attention to climate change, and social were perceived to be the most critical barriers. Even though these were highlighted, it is worth noting that the literature review highlighted that barriers differ from project to project, are highly context-specific and may be influenced by underlying causes.

Among the reasons that were cited were that in an environment where resources are limited, there will always be competition for resources. The long-term nature of adaptation projects or effects presents problems because adaptation is not prioritised now because its effects may only be felt much later. Accordingly, Ekins and Speck (2014), had stressed the need to reduce uncertainty of climate change outcomes because this makes it difficult to justify substantial expenditure today to gain uncertain benefits, perhaps in the distant future.

Furthermore, some of the benefits of adaptation policies may be in the form of co-benefits. Hence, the authors argue that it is crucial for policymakers when they make decisions, which have long-term implications, to understand as much as possible the full range of links between their current actions and future outcomes. The respondents felt that climate change is seen largely as the preserve of intellectuals and has not been simplified for ordinary people on the ground (Respondent 16).

**FIGURE 5:**
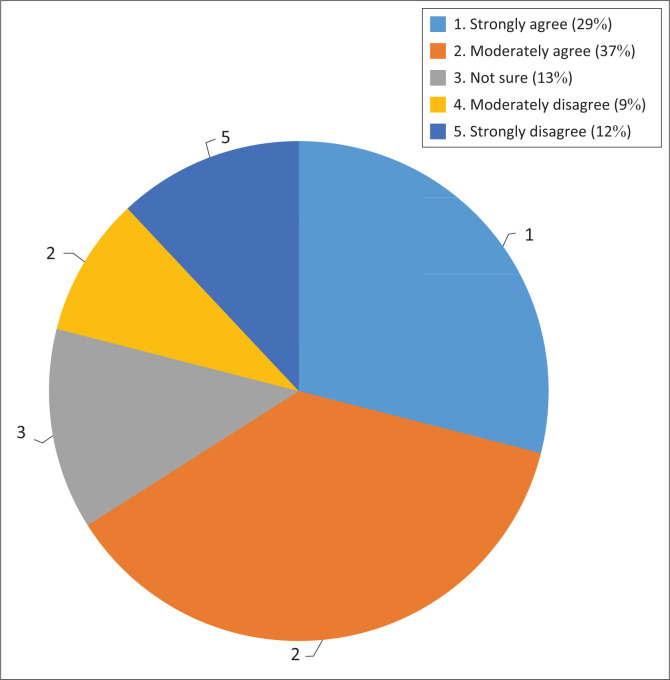
Balance between short- and long-term adaptation scenarios.

Even with intellectuals, it was contended that it seems that the understanding is largely limited to physical science and not full appreciation of what it means for specific sectors (Respondent 19). The respondents observed the fact that adaptation is viewed as costly, generally not bankable and profit-oriented, which is not helpful. They decried the lack of national attention to adaptation simply because it is not being taken seriously; hence there is no robust public awareness campaign on it. Conflicting timescale was also highlighted as a barrier, especially when one considers the fact that climate change competes with other issues for an already limited amount of political and economic attention that are more pressing in nature, whose impacts are more certain and more visible short-term results compared with adaptation that requires a long-term horizon. Faling et al. (2012) correctly acknowledged that government is torn between attending to pressing socio-economic development priorities. When asked if there is balance between the short-term adaptation benefits while at the same time addressing long-term adaptation scenarios, 66% of the respondents surveyed felt there was a balance between short- and long-term adaptation scenarios (Figure 5).

The respondents indicated that the effect of a political-term of office cannot be ignored when it comes to issues that get political attention, because inevitably, there will always be competition for priorities. Hence, Galvani (2018) correctly asserted that the extent of support or opposition to a policy also depends on the perceived electoral gains provided by a given policy. For instance, Respondent 2 suggested that even internationally, climate change is not prioritised at the level of the United Nations Security Council’s issues, which leaves climate change at the fringes.

**FIGURE 6:**
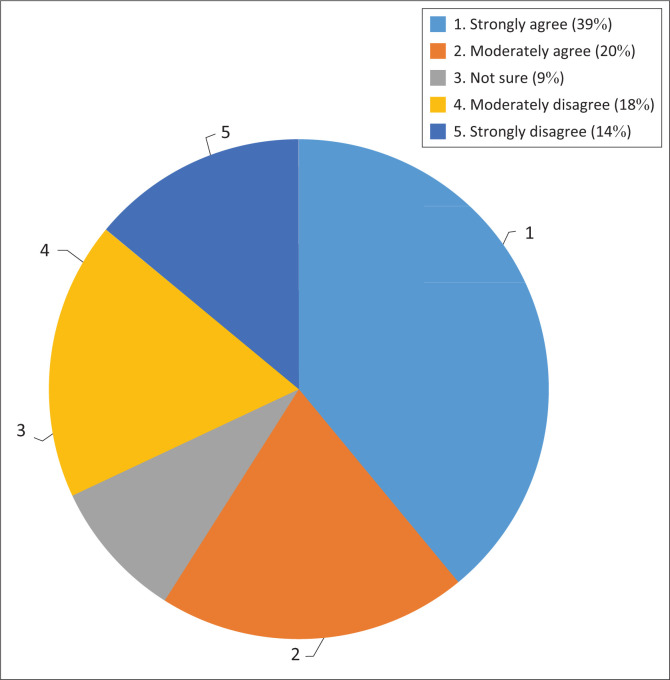
Adaptation should be addressed purely in the context of other programmes.

Respondents were also asked whether adaptation should be addressed purely in the context of other programmes such as disaster risk management, sustainable development and not as a standalone measure. A total of 59% felt that it should not be addressed in isolation (Figure 6). This view is consistent with the observation in the literature review, even though such an approach may unintentionally diminish the role of adaptation in policy and broader political discourse. The respondents argued that climate change needs a holistic process that is effective, efficient and not myopic taking into account resource constraints.

In supporting the notion that adaptation should be integrated into other programmes, it was argued that one cannot dissociate climate change from things such as disaster risk management, sustainable development and other issues were seen as two sides of the same coin (Respondent 19). Ekins and Speck (2014), expressed the same sentiment by suggesting that measures for adaptation to climate change may include among others early warning systems for storms, flood defences or changes to construction or infrastructure to ensure that they are able to withstand harsher impacts from changes in weather. For that reason, it was implied that it does not really matter whether the intervention is within the context of the SDGs or climate change. All that matters is the contribution to the broader good and how each of those actions are making progress in various matrixes that are used by the different international instruments (Respondent 19). Inevitably, whatever actions that are undertaken would have a common central nexus, which is sustainable development within which there is human well-being.

## Conclusion

The purpose of this article was to investigate and present the findings relating to policy coverage and institutional spread that are in place to address climate change adaptation capacity and resilience building. The study contributed to knowledge by examining adaptation policies, improving practice and even decision-making by government on adaptation measures. The study revealed that South Africa has policies and strategies in place even though it was inferred that they are superficial, difficult to implement and not supported by resources. The study demonstrated that part of the problem is the lack of leadership and a clear vision for adaptation. The study concluded that policies have not assisted to make South Africa to build climate change resilience. There was also a concern about poor institutional coordination. Although climate change adaptation is regarded as important in South Africa, there is a lack of emphasis on adaptation in general. Hence, adaptation is neglected. In the light of these challenges and considering South Africa’s development agenda, the study recommends that there should be deliberate enhancement of adaptation measures at all levels of government. Furthermore, a full alignment of the climate action goal is necessary, specifically the adaptation and resilience perspectives should be addressed within the context of other programmes such as disaster risk management and sustainable development.

## Footnotes

1 This is a structure consisting of the minister and the nine provincial Members of Executive Council (MECs) who deal with the same portfolio.

2 MINTEC is a structure consisting of senior government officials that was established to facilitate coordination between the national department and the provincial authorities particularly on environmental affairs. It supports MINMEC and is chaired by the director general.
